# Surface Roughness, Microhardness, and Microleakage of a Silorane-Based Composite Resin after Immediate or Delayed Finishing/Polishing

**DOI:** 10.1155/2016/8346782

**Published:** 2016-02-09

**Authors:** Fernanda Carvalho Rezende Lins, Raquel Conceição Ferreira, Rodrigo Richard Silveira, Carolina Nemésio Barros Pereira, Allyson Nogueira Moreira, Claudia Silami Magalhães

**Affiliations:** ^1^Department of Restorative Dentistry, School of Dentistry, Federal University of Minas Gerais, Avenida Antônio Carlos 6627, Pampulha, 31270-901 Belo Horizonte, MG, Brazil; ^2^Department of Social and Preventive Dentistry, School of Dentistry, Federal University of Minas Gerais, Avenida Antônio Carlos 6627, Pampulha, 31270-901 Belo Horizonte, MG, Brazil

## Abstract

*Objective*. This study evaluated the effect of immediate or delayed finishing/polishing using different systems on the surface roughness, hardness, and microleakage of a silorane-based composite.* Material and Methods*. Specimens were made with silorane-based composite (Filtek P90, 3M ESPE) and assigned to the treatments: control (light-cured); aluminum oxide discs (Sof-Lex, 3M ESPE); diamond-impregnated silicone tips (Astropol, Ivoclar Vivadent); aluminum oxide-impregnated silicone tips (Enhance, Dentsply). Half of the specimens were finished/polished immediately and the rest after 7 days. Surface roughness (Ra, *μ*m; *n* = 20) and Vickers microhardness (50 g; 45 s; *n* = 10) were measured. Cavities were prepared in bovine incisors and filled with Filtek P90. The fillings received immediate or delayed finishing/polishing (*n* = 10) and were subjected to dye penetration test (0.5% basic fuchsin, 24 h). Data were analyzed by ANOVA and Scheffe, Kruskal-Wallis, and Mann-Whitney tests (*p* < 0.05).* Results*. The finishing/polishing system significantly influenced roughness and microhardness (*p* < 0.0001). For enamel, microleakage was not affected by the finishing/polishing system (*p* = 0.309). For dentin, Sof-Lex discs and Astropol points promoted greater microleakage than Enhance points (*p* = 0.033).* Conclusion*. Considering roughness, microhardness, and microleakage together, immediate finishing/polishing of a silorane-based composite using aluminum oxide discs may be recommended.

## 1. Introduction

Dental restorations require finishing and polishing procedures to fulfill the requirements of form and function and to promote aesthetics, longevity, and periodontal health [1, 2]. Different methods can be used for finishing and polishing, and the surface smoothness obtained is dependent on the composition of the composite, the presence of bubbles, and the instruments and procedures used [3].

There is controversy regarding the best time to perform finishing and polishing of composite restorations. Although composite manufacturers recommend performing the procedure immediately after the restoration, it should be delayed to avoid the negative effects of heat generation, such as smearing of the resin matrix and the creation of local hot spots before the final polymerization of the composite [4]. However, delayed finishing and polishing resulted in lower microhardness than immediate polished composite specimens [5].

A silorane-based low-shrinkage composite has been introduced as an alternative to methacrylates and has low filler content by volume with a combination of fine quartz particles and yttrium fluoride [6]. When compared to methacrylate-based resins, the silorane-based composite has exhibited lower water sorption and solubility [7], lower adhesion potential of oral streptococci, and similar adhesion potential of* Candida albicans *[8–10]. Cusp deflection caused by polymerization contraction was significantly lower on teeth restored with an experimental silorane-based composite compared to teeth restored with a methacrylate-based composite [11]. Furthermore, no leakage was found when mesio-occluso-distal cavities were restored with silorane-based composites [12]. Silorane has shown lower compressive strength and microhardness and greater flexural strength and fracture toughness [13]. However, silorane has shown lower degrees of conversion and polymerization depth than methacrylate-based composites [14].

Changes in matrix composition, the introduction of new monomers, filler content optimization, and variations in particle size, type, and morphology can all increase the surface roughness of composites [15] and can result in plaque accumulation, gingival inflammation, and surface staining [16]. Few previous studies have compared the use of different finishing and polishing systems on a silorane-based composite [15, 17] and, to date, no studies have verified the effect of immediate or delayed polishing on this restorative material.

The objective of the present study was to evaluate the effect of immediate or delayed finishing/polishing using different systems on the surface roughness, hardness, and microleakage of a silorane-based composite. The null hypothesis tested was that the time to perform finishing/polishing and the use of different systems do not affect the surface roughness, microhardness, and microleakage of a silorane-based composite resin.

## 2. Material and Methods

### 2.1. Experimental Design

The main factors evaluated in this* in vitro *study were finishing/polishing systems at four levels—control (light-cured in contact with polyester strip), aluminum oxide discs (Sof-Lex, 3M ESPE), diamond-impregnated silicone tips (Astropol, Ivoclar Vivadent), and aluminum oxide-impregnated silicone tips (Enhance, Dentsply)—and the time to perform finishing/polishing at two levels (immediately and after 7 days). The specimens were made of silorane-based composite (Filtek P90, 3M ESPE) following a randomized complete block design. The dependent variables were mean surface roughness (Ra, *μ*m) (*n* = 20), Vickers microhardness (*n* = 10), and microleakage at the enamel and dentin margins, evaluated by dye penetration scores (*n* = 10). The surfaces of the specimens were analyzed using scanning electron microscopy (SEM).  Table 1 shows the composition, batch, and manufacturer of the studied materials.

### 2.2. Specimen Fabrication

A two-part Teflon mold (diameter = 10 mm and high = 2 mm) was filled with the composite in a single increment, using a 70 spatula (SS White/Duflex, Rio de Janeiro, RJ, Brazil). A polyester strip and a glass slide were positioned on the strip, and a load of 500 g was applied for 30 seconds, followed by light-curing (40 seconds, 600 mW/cm^2^) using a QHT light-cure unit (Demetron LC, Kerr Corporation, Middleton, WI, USA) positioned in direct contact with the polyester strip. A marking was made on the outer edge of each specimen to standardize the direction of rotating device application. Half of the specimens of each group randomly divided received finishing/polishing immediately after preparation. The other half was stored in dark container on gauze soaked in distilled water at 37°C for 7 days. The following finishing/polishing procedures were applied.

*Control*. No procedure was performed. The surface finish was provided by the polymerization of the composite in contact with a polyester strip.

*Sof-Lex*. It is the sequential application of medium, fine, and superfine grain discs, mounted on the handpiece. Each disc was applied to the specimen for 10 seconds under constant cooling with a water jet. Irrigation was performed between each application with compressed air/water for 5 seconds. One disc sequence was used for each specimen. 

*Enhance*. It is the application of disc-shaped Enhance tips, mounted on the handpiece for 30 seconds under constant cooling with a water jet. One tip was used for each specimen. 

*Astropol*. This is the sequential application of Astropol HP (gray), Astropol P (green), and Astropol F (pink) discs, mounted on the handpiece. Each disc was applied to the specimen for 10 seconds under constant cooling with a water jet. Irrigation was performed between each application with compressed air/water for 5 seconds. One disc sequence was used for each specimen.

After finishing and polishing, the specimens were washed for 10 seconds with compressed air/water and cleaned (ultrasonic bath for 30 seconds in distilled water), dried with paper towels, and stored dry. A single operator performed all laboratory procedures in an air-conditioned environment at temperature of 21 ± 2°C. The same specimens were used sequentially for measurements of surface roughness and microhardness.

### 2.3. Surface Roughness

A profilometer (Mitutoyo SJ-301 Surftest, Aurora, IL, USA) calibrated with a standard of known roughness was used. The arithmetic mean of the absolute distance of the roughness profile (Ra, *μ*m) was recorded within a measuring length of 4 mm and with a cut-off of 0.8 mm. Four readings were taken for each specimen, one parallel, one perpendicular, and two diagonal in relation to the direction of the finishing/polishing instrument application. The mean of the four readings was obtained to represent each specimen.

### 2.4. Microhardness

Vickers microhardness was conducted on a MVK-H1 microhardness tester (Hardness Testing Machine, Mitutoyo, Kanagawa, Ken, Japan) under 50 g load, over 45 seconds. Four indentations were made on each specimen, one in each quadrant, equidistant from the center. The readings were recorded immediately after removal of the penetrator to minimize the effect of elastic recovery. The mean of the four indentations was used to determine the Vickers hardness number of each specimen.

For illustrative purposes, two specimens representative of each experimental condition were prepared for surface characterization using SEM (Quanta 200F, FEI, Hillsboro, OR, USA). The specimens were metallized with carbon, and the surface was examined with up to 3,000x magnification and an accelerating voltage of 10,000 V.

### 2.5. Restoration of Cavities

Extracted bovine teeth were scaled to remove organic and inorganic debris and were stored in 0.05% thymol solution. Eighty teeth were visually selected based on the absence of cracks, stains, and excessive incisal wear. After thorough washing in running water, cylindrical cavities (2.0 mm in diameter and 1.5 mm in depth) were prepared using 2294 diamond bur (KG Sorensen, Cotia, SP, Brazil) in a high-speed handpiece under constant air/water cooling. Two cavities were prepared on the buccal surface of each tooth to 3 mm of the enamel-cement junction, one on the coronal portion and the other on the root surface. A new diamond tip was used for every two prepared teeth. The cavities dimensions were checked using a millimeter probe (Hu-Friedy, Chicago, IL, USA). The coronal restoration operative steps were (a) application of 37% phosphoric acid (Condac, FGM, Joinville, SC, Brazil) for 30 seconds on the enamel, washing with air/water spray for 60 s, and drying with paper towels; (b) active priming (Silorane Adhesive System, 3M ESPE) application for 15 seconds, applying a gentle air stream until the primer was spread into a uniform film, and light-curing for 10 seconds; and (c) adhesive application (Silorane Adhesive System, 3M ESPE) into the cavity, applying a gentle stream of air until the adhesive was spread into a uniform film, and light-curing (10 seconds). The composite was then inserted into the cavity in a single increment and was light-cured (40 seconds, 600 mW/cm^2^) in contact with a polyester strip. The gross excesses were removed with a 15 scalpel blade.

Root cavities were restored following the operative steps described above, except for the application of phosphoric acid.

### 2.6. Restoration Finishing and Polishing

The restorations were subjected to the same finishing/polishing procedures as described for the roughness and microhardness tests.

Half of the specimens received finishing/polishing immediately after preparation. The other half were stored in a dark container on a gauze pad soaked in distilled water at 37°C and received finishing/polishing seven days after preparation. A single operator performed all laboratory procedures in an air-conditioned environment at a temperature of 21 ± 2°C.

### 2.7. Microleakage Test

An adhesive tape disc (Scotch Magic Tape® 3M Ltda., São Paulo, SP, Brazil) 3 mm in diameter was applied to the restoration and adjacent tooth structure. The apical foramen was covered with plastified wax (Wilson, Polidental, Cotia, SP, Brazil), and a double layer of nail polish (Impala, Mundial SA, São Paulo, SP, Brazil) was applied over the entire tooth surface.

After the nail polish dried, the tape discs were removed to expose the restoration and the 1 mm adjacent tooth structure. The specimens were immersed in 0.5% basic fuchsin for 24 hours at room temperature, washed in running water for 2 minutes, and dried with absorbent paper. The teeth were sectioned on the enamel-dentin junction to separate the crowns of the roots. Then they were sectioned longitudinally in a buccolingual direction through the center of the restorations using a double-faced diamond disc (KG Sorensen, Cotia, SP, Brazil).

A trained examiner (Kappa = 0.86) examined both crown and root hemisections to determine the scores of dye penetration in the restoration margins. The cervical and incisal or apical margins of the coronal and root restorations were examined separately using a stereomicroscope (Stemi DV4, Carl Zeiss, Göttingen, LS, Germany) with 32x magnification and were classified according to the following scores: (0) no evidence of dye penetration at the tooth/restoration interface; (1) dye penetration up to half the length of the wall; (2) dye penetration along all the length of the wall; and (3) dye penetration along all the length of the wall and in the axial direction.

### 2.8. Statistical Analysis

Kolmogorov-Smirnov and Shapiro-Wilk tests verified the normal distribution of surface roughness (*p* = 0.094) and microhardness data (*p* = 0.200). Levene's test was used to verify the equality of variances for the surface roughness (*p* = 0.547) and microhardness (*p* = 0.916) data. The effects of the finishing and polishing systems, the time of application, and the interactions were analyzed using two-way Analysis of Variance (ANOVA) and Scheffe* post hoc* test. Kruskal-Wallis test compared median microleakage scores to determine the effects of finishing/polishing systems and time of application on the enamel and dentin margins. Comparison of the polishing systems was performed using the Mann-Whitney test. All tests were considered significant at 95% confidence level.

## 3. Results

Two-way ANOVA found a significant effect of finishing/polishing system on surface roughness (*p* < 0.0001). There was no significant effect of the application time (*p* = 0.2851) or the interaction between polishing system and application time (*p* = 0.0669). The control group produced significantly less surface roughness than the other types of polishing, which did not differ from each other (Table 2).

Two-way ANOVA showed a significant effect of finishing/polishing system on microhardness (*p* < 0.0001). There was no significant effect of the application time (*p* = 0.500) or of the interaction (*p* = 0.313). The control group had significantly lower microhardness than the Astropol and Sof-Lex groups but did not differ from the Enhance group. Enhance did not differ from Astropol but produced significantly lower microhardness than Sof-Lex. Sof-Lex produced significantly higher microhardness than control and Enhance but did not differ from Astropol (Table 3).

For the enamel margins, delayed polishing produced significantly higher microleakage than immediate polishing (*p* = 0.009), but there was no significant effect of finishing/polishing system (*p* = 0.309). Medians, minimum and maximum values, and mean ranks of microleakage observed for immediate and delayed finishing/polishing in enamel and dentin are presented in Table 4.

For the dentin margins,there was nosignificant effect of the application time (*p* = 0.313), but there was a significant effect of finishing/polishing type on microleakage (*p* = 0.033).  Table 5 shows median, minimum and maximum values, and mean ranks of microleakage for the finishing/polishing systems studied.

**Figure 1:**
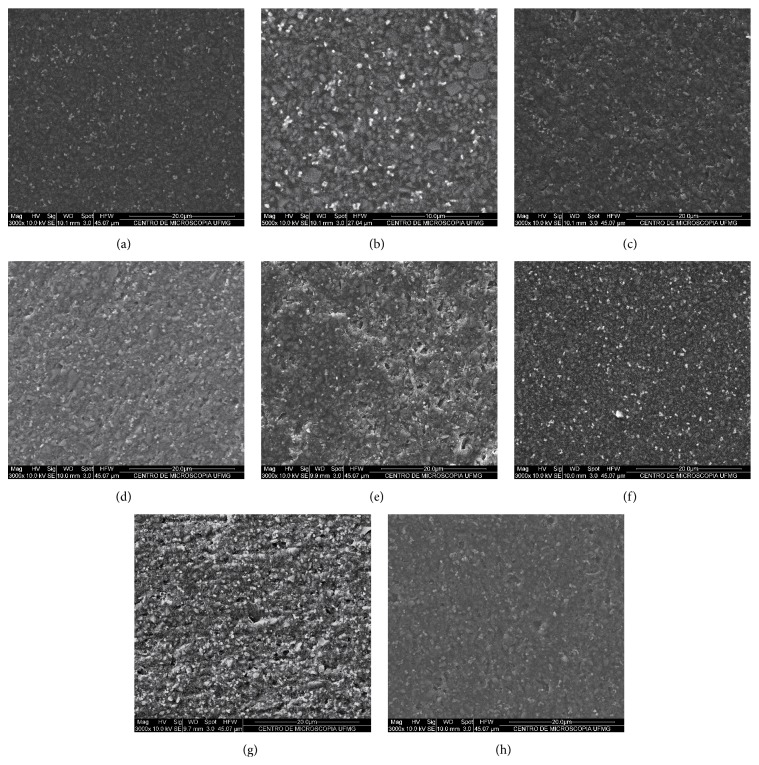
SEM images of silorane-based composite resin surface: (a) control, without surface finishing and polishing 3,000x magnification and (b) 5,000x magnification; (c) surface finished and polished with the Astropol immediately and (d) after 7 days (3,000x); (e) surface finished and polished with Enhance system immediately and (f) after 7 days (3,000x); and (g) surface finished and polished with Sof-Lex discs immediately and (h) after 7 days (3,000x).

SEM analysis showed that when subjected to immediate or delayed finishing/polishing procedures, the composite surface showed more irregularities than the control. The control group exhibited a smoother texture and unscratched surface (Figures 1(a) and 1(b)). Scratches and porosity resulting from the displacement of particles were observed on the surfaces treated with Astropol (Figures 1(c) and 1(d)), Enhance (Figures 1(e) and 1(f)), and Sof-Lex discs (Figures 1(g) and 1(h)). In agreement with the profilometric findings, rougher surfaces were observed in the groups that underwent finishing and polishing, with no differences between them and between times of application.

## 4. Discussion

Different methods can be used for the finishing and polishing of restorative composites. The polyester strip provides a smoother surface, but its use is limited by the complex occlusal anatomy and need for functional adjustments in almost all restorations, requiring the use of tools to provide a smooth final surface [5].

Surface roughness is a property resulting from the interaction of many factors. Some of these factors are intrinsic to the material and are related to its composition, such as filler type, shape, size, and distribution, the type of resin matrix, the degree of final cure achieved, and the bond efficiency at the filler/matrix interface. Extrinsic factors are associated with the type of polishing system used, such as the flexibility of the material in which the abrasives are incorporated, the hardness of the abrasives, the geometry of the instruments, and the way they are used [3, 17, 18].

In the present study, the surface roughness obtained for the silorane-based composite light-cured in contact with a polyester strip (0.193 ± 0.063 *μ*m) was similar to the mean found in a previous study (0.220 ± 0.292) [1]. These values are close to the critical value of 0.2 *μ*m proposed as the roughness threshold required for the accumulation of biofilm [2]. Silorane-based composite presented comparable or less roughness than microhybrid and nanoparticle composites for restorations in posterior teeth when subjected to standardized polishing with 1000 grade abrasive sandpaper [2, 3, 13, 19]. The relatively low quartz and yttrium fluoride particle contents (76% p/p or 55% p/v) and the mean particle size of 0.5 *μ*m (0.1–2 *μ*m) in silorane-based composite may have contributed to this surface roughness value [13, 14, 20]. The images obtained by SEM confirmed these findings, showing a smooth surface texture without scratches in the control group.

However, in this study, after the application of the finishing and polishing systems, the resulting mean roughness in all groups was approximately more than twice the value of 0.2 *μ*m for both immediate and delayed polishing. The mean values measured were higher than those shown in other studies [3, 8, 10, 18, 21]. Some studies employed 1000 to 4000 grade sandpaper in a mechanical polisher with water lubrication and controlled rotation before the application of the polishing systems under testing conditions [3, 8, 10, 18]. This procedure may have favored to obtain a lower mean Ra because it promotes more uniform removal of the surface layer of the composite.

In the present study, no statistically significant difference was observed between the systems studied regarding surface roughness. This result is in agreement with another study that compared different finishing and polishing systems for silorane-based composites [18]. No significant difference was observed for the application times of finishing and polishing procedures, demonstrating that immediate or delayed polishing did not affect this property. This behavior has been previously described for microparticulate and hybrid methacrylate-based composites [4, 5, 22]. Images obtained by SEM showed scratches and porosities resulting from particle displacement on the surfaces submitted to finishing and polishing instruments at both application times.

To ensure the effectiveness of the finishing system, the abrasive grain particles should be harder in relation to the filler particles [18]. The present study showed similar performance between aluminum oxide discs, diamond-impregnated silicone tips, and silicone discs covered with aluminum oxide. Different effects could be expected because Astropol HP contain diamond particles in its composition, while Sof-Lex discs and Enhance tips use aluminum oxide as abrasive particles. Diamond is harder than aluminum, causing deeper grooves on the surface of the composite, which results in more roughness [18]. Nonetheless, instruments impregnated with aluminum oxide were able to wear quartz and yttrium fluoride particles and the silorane matrix uniformly [17, 18, 21].

Hardness can be defined as the resistance of solid structures to permanent indentation or penetration. Changes in hardness may reflect the cure state of a material and the presence of either a continuous reaction or the maturity of the restorative material [4, 5, 15, 22]. In general, increasing the particle size increases the resistance and surface hardness of the composite. Moreover, the type, morphology, distribution, and volume fraction of filler particles and the concentration of diluent monomers affect the hardness of the composite [15, 23].

The Vickers hardness number (VHN) reported for silorane-based resin (measured after polishing with abrasive sandpaper) has ranged from 59.26 to 80.8 [10, 18, 20, 24]. In this study, the mean VHN measured immediately after the use of different finishing and polishing systems ranged from 55.77 to 60.91. This lower range may be related to the fact that no prior polishing with abrasive sandpaper was performed to mimic the clinical conditions in which finishing and polishing systems are responsible for the removal of the surface layer rich in organic matrix.

In this experiment, the use of different finishing and polishing systems resulted in a significant difference in the microhardness of silorane-based resin when it was applied either immediately or after 7 days. The use of Sof-Lex resulted in significantly higher microhardness compared to Enhance, which was not different from Astropol, which yielded intermediate microhardness values. We did not find previous studies that have evaluated the microhardness of silorane-based resin in response to different finishing and polishing systems. It is likely that the use of the abrasive disc sequence more effectively removed the organic matrix rich layer compared to other systems, exposing a harder surface. The different finishing and polishing systems showed the same behavior after 7 days. One might expect to find greater hardness for a silorane-based composite polished after 7 days because its polymerization reaction is characterized by continuous cationic ring opening initiated at the time of light-curing [15]. However, no significant differences were observed in the hardness of the silorane-based resin after various storage times (1 to 30 days), probably due to the presence of siloxane, which has reduced water solubility and sorption [7].

The dye penetration test was performed to determine the marginal sealing capacities of the restorations under the conditions evaluated. Overall, median microleakage scores were low (0.00 to 0.50) for the enamel and dentin margins. Silorane-based composite restorations of Class II and Class V cavities with enamel and dentin margins had similarly low microleakage results [13, 19, 25–29]. This result can be attributed to the ring opening chemical of the silorane system, which provides the system with less polymerization shrinkage, and to the enamel and dentin adhesion promoted by the self-etching Silorane Adhesive System.

The effects of the finishing and polishing systems on microleakage were observed only for the dentin substrate. The use of the Sof-Lex discs promoted greater microleakage compared to the control and Enhance and did not differ from Astropol. It seems that the use increasing grit sequential tips or discs produced some kind of damage to the tooth-restoration union, notably on the dentin margins, which have less mineral content and more moisture compared to the enamel. Delayed polishing promoted greater microleakage than immediate polishing, only on the enamel margins. It suggests that after 7 days of storage in a humid environment at 37°C, there was a loss of bond quality of the self-etching adhesive system, even when enamel etching had previously been performed with 37% phosphoric acid. Immediate polishing did not make the silorane-based composite more susceptible to the supposed adverse effects of heat generation, probably due to the stability of its aromatic rings [6].

## 5. Conclusion

Considering the limitations of this* in vitro* study, it can be concluded that immediate polishing does not adversely influence the surface roughness, microhardness, and microleakage of silorane-based composite resin. The sequential aluminum oxide discs system produced a beneficial effect on microhardness but negatively influenced microleakage on the dentin margins. Considering the three outcomes studied as a whole, no finishing and polishing system performance was superior to the others.

## Figures and Tables

**Table 1:** Compositions, batches, and manufacturers of the studied materials.

	Composition	Batch number	Manufacturer
Sof-Lex	Aluminum oxide (medium: 40 *µ*m, fine: 24 *µ*m, x-fine: 8 *µ*m)	1202000341	3 M/ESPE Dental Products, Seefeld, Bavaria, Germany

Astropol	Caoutchouc, silicon carbide, aluminum oxide, titanium oxide, and iron oxide (coarse gray (45 *µ*m), fine green (1 *µ*m), Caoutchouc, silicon carbide, aluminum oxide, titanium oxide, and Diamond dust 9 extra-fine-pink (0.3 *µ*m))	11361 12893 13264	Ivoclar Vivadent, Schaan, Liechtenstein

Enhance	Aluminum oxide, silicon dioxide finishing wheel-impregnated UDMA (45 *µ*m)	657903E	Dentsply, Petrópolis, RJ, Brasil

Filtek P90	Yttrium fluoride, 3,4 Epoxycyclohexaylcyclopoly-methylsiloxane, silorane, siloxane, silanized quartz Filler size (0.1–2 *µ*m), 76% (p/p) ou 55% (p/v)	1227700149 1303700268	3 M/ESPE, St. Paul, MN, EUA

Silorane Adhesive System	Self-etching primer: phosphorylated methacrylates, Vitrebond™ copolymer, BisGMA, HEMA, water, ethanol, silane treated silica, initiator, and stabilizer Adhesive: hydrophobic bifunctional monomer, acidic monomers, a silane-treated silica, initiator, and stabilizer	1109400719 1221900749	3 M/ESPE, Seefeld, Bavaria, Germany

Condac	Phosphoric acid 37%, thickener, dye, and deionized water	25012013	FGM, Joinville, SC, Brasil

**Table 2:** Comparison of roughness (Ra, *µ*m) means (± standard deviation) for the finishing and polishing systems studied according to application time.

	Control	Sof-Lex	Astropol	Enhance
Immediate	0.1933^aA^	0.4788^^bA^^	0.3803^bA^	0.4938^bA^
	(±0.0636)	(±0.1555)	(±0.1406)	(±0.1271)

Delayed	0.1913^aA^	0.4191^bA^	0.4365^bA^	0.4162^bA^
	(±0.0594)	(±0.1004)	(±0.1425)	(±0.1629)

Means followed by different lowercase letters show statistically significant differences between them, as compared in rows.

Means followed by the same capital letters do not show statistically significant differences between them,  as compared in columns.

**Table 3:** Comparison of Vickers microhardness means (± standard deviation) for each finishing and polishing systems studied according to application time.

	Control	Sof-Lex	Astropol	Enhance
Immediate	50.59^Aa^	60.91^Ac^	55.77^Abc^	55.95^Aab^
	(±5.55)	(±8.90)	(±7.30)	(±6.83)

Delayed	53.88^Aa^	59.56^Ac^	58.13^Abc^	54.65^Aab^
	(±3.28)	(±6.04)	(±4.92)	(±4.26)

Means followed by different lowercase letters show statistically significant differences between them,  as compared in rows.

Means followed by the same capital letters do not show statistically significant differences between them, as compared in columns.

**Table 4:** Comparison of medians (minimum–maximum) and mean ranks of microleakage according to application time, in enamel and dentin margins.

	Enamel margins		Dentin margins	
	Median (min–max)	Mean rank	Median (min–max)	Mean rank
Immediate	0.00 (0.00–3.00)	34.18^a^	0.00 (0.00–3.00)	38.10^a^

Delayed	0.50 (0.00–3.00)	46.83^b^	0.50 (0.00–3.00)	42.90^a^

^*∗*^Values followed by different letters show statistically significant differences between them, as compared in columns.

**Table 5:** Comparison of medians (minimum–maximum) and mean ranks of microleakage according to the different finishing and polishing systems studied, in the dentin margins.

	Median (min–max)	Mean rank
Control	0.000 (0.00–2.00)	34.63^ac^
Enhance	0.000 (0.00–2.00)	32.65^a^
Astropol	0.500 (0.00–3.00)	45.53^bc^
Sof-Lex	0.500 (0.00–3.00)	49.20^b^

^*∗*^Values followed by different letters show statistically significant differences between them.
